# Cerebrospinal Fluid Transforming Growth Factor β Isoforms and Disease Progression in Alzheimer’s Disease: Longitudinal Evidence from the ADNI Cohort

**DOI:** 10.3390/neurolint18070138

**Published:** 2026-07-20

**Authors:** Manal Aljuhani, Azhaar Ashraf, Abdullah Alqarni, Mohammed S. Alshuhri, Essam Mohammed Alkhybari, Amani Alharbi, Alanoud Almudayni, Fatmah Jamal Alablani, Azhar Akhmimi, Ahmad A. Alhulail

**Affiliations:** 1 Department of Radiology and Medical Imaging, College of Applied Medical Sciences, Prince Sattam Bin Abdulaziz University, Al-Kharj 16278, Saudi Arabia; 2 Radiological Sciences and Technology Research Unit, College of Applied Medical Sciences, Prince Sattam Bin Abdulaziz University, Al-Kharj 16278, Saudi Arabia; 3 Division of Neurology, Department of Brain Sciences, Faculty of Medicine, Imperial College London, London W12 0NN, UK; 4 Department of Cardiovascular Technology-Echocardiography, College of Applied Medical Sciences, King Saud Bin Abdulaziz University for Health Sciences, Riyadh 11481, Saudi Arabia; 5 King Abdullah International Medical Research Centre, Riyadh 11481, Saudi Arabia

**Keywords:** ADNI, Alzheimer’s disease, amyloid β, cerebrospinal fluid biomarkers, disease progression, FDG-PET, neuroinflammation, regression, tau, transforming growth factor β

## Abstract

**Background:** The role of cerebrospinal fluid (CSF) transforming growth factor β (TGF-β) isoforms in Alzheimer’s disease (AD) remains unclear. We examined associations of CSF TGF-β1, TGF-β2, and TGF-β3 with AD biomarkers, neurodegeneration, cognition, and clinical progression. **Methods:** In 294 ADNI participants with baseline CSF TGF-β measurements, adjusted regression, and mixed-effect models were used to evaluate associations with CSF biomarkers, neuroimaging, cognitive outcomes, and conversion. False-discovery-rate correction was applied. **Results:** Higher baseline TGF-β1 was associated with higher CSF total tau (β = 52.68 pg/mL per 1 SD increase; *q* < 0.001) and p-tau (β = 5.68 pg/mL; *q* < 0.001). Higher TGF-β2 was associated with faster hippocampal volume loss (β = −45.42 mm^3^/year; *q* < 0.001). No isoform was robustly associated with FDG-PET decline, cognitive decline, clinical conversion, or time to conversion. **Conclusions:** CSF TGF-β1 and TGF-β2 show distinct associations with tau-related pathology and hippocampal neurodegeneration, respectively, but do not appear to be prognostic biomarkers of clinical progression in AD.

## 1. Introduction

Alzheimer’s disease (AD) is a progressive neurodegenerative disorder and the most common cause of dementia worldwide. Despite major advances in biomarker development and disease definition, AD remains clinically heterogeneous, with substantial variability in rates of cognitive decline and disease progression among individuals [1]. While amyloid β (Aβ) and tau biomarkers are well established for diagnosis and staging, they do not fully capture the biological mechanisms that govern disease trajectory [2,3]. Identifying biomarkers that reflect downstream pathological processes influencing progression remains a critical unmet need [4,5].

Neuroinflammation is increasingly recognised as a central component of AD pathophysiology and a key modifier of disease progression [6]. Beyond being a secondary response to protein aggregation, immune and glial responses are now understood to actively shape neuronal vulnerability and synaptic dysfunction [7]. The glial cell dysregulation hypothesis proposes that dyshomeostasis of microglia and astrocytes contributes fundamentally to the development and progression of AD [8]. Microglia act as a double-edged sword, exerting both neuroprotective and cytotoxic effects depending on their activation state, while astrocytes play a critical regulatory role in shaping microglial behaviour [9,10].

A central mediator of astrocyte–microglia crosstalk is transforming growth factor β (TGF-β), a multifunctional cytokine family with essential roles in immune regulation within the central nervous system [11,12]. Astrocytes secrete TGF-β, which in turn modulates microglial cytotoxicity, phagocytic activity, cytokine production, and reactive oxygen species release [11]. TGF-β exists in three isoforms—TGF-β1, TGF-β2, and TGF-β3—which share overlapping, but distinct biological functions [13,14,15,16]. Experimental studies have implicated TGF-β signalling in the modulation of neurodegeneration and AD-like pathology, influencing processes such as amyloid clearance, blood–brain barrier integrity, and glial activation states. However, the specific roles of individual TGF-β isoforms during the early stages of AD and across the disease continuum remain poorly defined.

Meta-analytic and experimental evidence indicates elevated TGF-β1 levels in AD biofluids and links astrocytic TGF-β overexpression to Aβ pathology, vascular dysfunction, and impaired cognition [17,18,19]. Conversely, loss of neuronal TGF-β signalling is associated with increased Aβ accumulation, synaptic degeneration, and neurodegeneration in both animal models and human AD brain tissue. Mechanistically, disrupted TGF-β/Smad signalling in neurons—partly due to sequestration by hyperphosphorylated tau—may impair neuroprotective responses. Together, these findings suggest that TGF-β signalling exerts both pathogenic and protective effects in AD depending on cellular source, disease stage, and signalling context [17,18,19].

Among the TGF-β isoforms, TGF-β2 is of particular interest due to its high expression in the brain and its involvement in neuronal–glial signalling [20]. Dysregulated TGF-β signalling has been linked to chronic neuroinflammatory states and impaired homeostatic responses, suggesting that altered levels may reflect maladaptive glial regulation rather than classical pro-inflammatory activation. Chong et al. (2017) reported associations between TGF-β2 and key neuropathological features of neurodegenerative disease, including neurofibrillary tangle burden, Lewy body pathology within Lewy body dementia, overall dementia severity, and levels of soluble Aβ_42_, suggesting a potential link between TGF-β2 signalling and disease-related pathological processes [20]. Despite this mechanistic relevance, few studies have examined CSF TGF-β isoforms as potential biomarkers of disease progression in AD, and their relationship with established AD biomarkers, neuroimaging measures, and cognition remains unclear.

The present study examined baseline CSF TGF-β1, TGF-β2, and TGF-β3 concentrations in participants from the Alzheimer’s Disease Neuroimaging Initiative. We tested their cross-sectional associations with CSF Aβ42, total tau, p-tau, FDG-PET, hippocampal volume, and cognitive measures, as well as their associations with longitudinal trajectories of CSF biomarkers, neuroimaging, and cognition. We also evaluated whether baseline TGF-β isoforms were associated with clinical conversion and time to conversion. By analysing all three isoforms using prespecified parallel models, this study aimed to clarify whether CSF TGF-β signalling is associated with distinct aspects of AD-related pathology, neurodegeneration, or clinical progression.

## 2. Materials and Methods

### 2.1. ADNI Study

#### 2.1.1. Study Design and Data Source

This study was a secondary analysis of longitudinal data from the Alzheimer’s Disease Neuroimaging Initiative (ADNI). The ADNI is a multicentre observational programme that collects clinical, cognitive, neuroimaging, genetic, and fluid-biomarker data across the AD continuum. The design of the ADNI cohorts, participant recruitment, clinical diagnostic procedures, imaging acquisition, cerebrospinal fluid processing, and assay quality-control procedures have been described previously (http://adni.loni.usc.edu/) [21], and included in the Supplementary Materials and Methods.

Data from ADNI-1 and ADNI-GO were used because baseline cerebrospinal fluid concentrations of TGF-β1, TGF-β2, and TGF-β3 were available in these datasets. The present analyses used de-identified secondary data. No new laboratory, imaging, or neuropsychological measurements were performed.

#### 2.1.2. Measures

Baseline CSF TGF-β1, TGF-β2, TGF-β3, Aβ42, total tau, and phosphorylated tau were obtained from ADNI biomarker data files. FDG-PET standardised uptake value ratio, hippocampal volume, ADAS-Cog 13, and RAVLT immediate recall were obtained from the corresponding ADNI data files. Detailed information on how the measures were collected by the ADNI are reported in the Supplementary Materials and Methods.

#### 2.1.3. Covariates and Data Handling

Age, sex, APOE ε4 carrier status, baseline diagnostic group, and baseline CSF Aβ42 were selected before analysis as covariates. Models with Aβ42 as the outcome were adjusted for age, sex, APOE ε4 carrier status, and baseline diagnostic group. Models with total tau or p-tau as outcomes additionally included baseline Aβ42. Hippocampal volume models also included intracranial volume. All TGF-β concentrations were standardised such that regression coefficients, odds ratios, and hazard ratios reflect a 1-standard-deviation increase in the relevant isoform.

Values recorded at the assay boundaries were retained at their reported limits in the primary CSF biomarker analyses: Aβ42 values >1700 pg/mL were coded as 1700 pg/mL; total tau values <80 pg/mL or >1300 pg/mL were coded as 80 pg/mL and 1300 pg/mL; and p-tau values <8 pg/mL or >120 pg/mL were coded as 8 pg/mL and 120 pg/mL. A sensitivity analysis excluded observations recorded at the assay boundaries.

### 2.2. Statistical Analyses

Data analysis was performed using Prism version 11.0.2 (GraphPad Software, CA, USA), SPSS version 32 (IBM Corp., Armonk, NY, USA), and R statistical software (R Foundation for Statistical Computing, Vienna, Austria). Continuous variables were summarised using means ± standard deviation or medians and interquartile ranges as appropriate, and categorical variables were summarised as counts and percentages. Distributional assumptions were evaluated using Shapiro–Wilk tests, histograms, quantile–quantile plots, and regression-residual diagnostics. Homogeneity of variance across diagnostic groups was evaluated using Levene’s test.

Since the three TGF-β isoforms were non-normally distributed, unadjusted diagnostic-group comparisons used Kruskal–Wallis tests. Associations between each isoform and each baseline CSF biomarker were estimated in separate ordinary least-squares regression models with HC3 robust standard errors. For each longitudinal outcome, a linear mixed-effect model was fitted with participant-specific random intercepts and random slopes for time. Fixed effects included time from baseline, baseline TGF-β isoform concentration, time-by-isoform interaction, and the prespecified covariates. The time-by-isoform interaction was the primary parameter for longitudinal analyses.

Clinical conversion status was evaluated using multivariable logistic regression. Time to conversion was evaluated using Cox proportional-hazard models stratified by baseline diagnostic group and adjusted for age, sex, APOE ε4 carrier status, and baseline Aβ42. The proportional-hazard assumption was examined using Schoenfeld-residual diagnostics. Kaplan–Meier curves were used as unadjusted descriptive analyses after dichotomizing each isoform at its median baseline concentration. TGF-β1, TGF-β2, and TGF-β3 were treated as prespecified candidate biomarkers and analysed in parallel. *p* values were adjusted using the Benjamini–Hochberg FDR procedure within each analysis family. Two-sided *p* values and adjusted *q* values are reported, with *q* < 0.05 considered statistically significant.

## 3. Results

### 3.1. Participants and Analysis Sets

**Figure 1:**
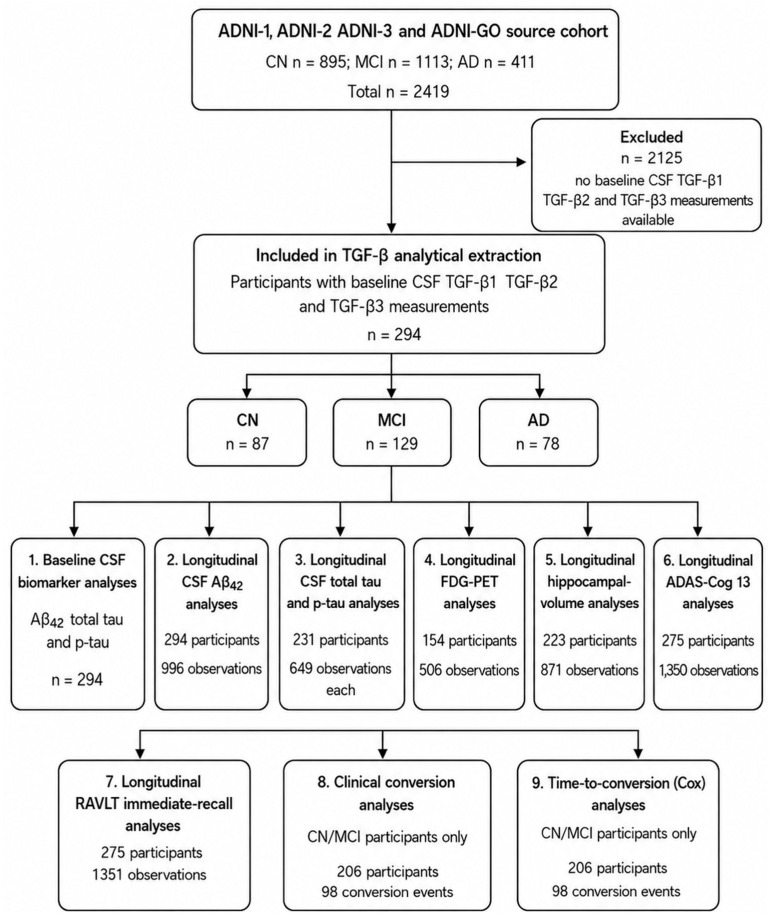
Cohort selection and outcome-specific analysis samples. The baseline analytical extraction included 294 participants with baseline CSF TGF-β1, TGF-β2, and TGF-β3 measurements. The chart shows the number of participants and repeated observations used for each outcome. Clinical conversion analyses were limited to cognitively normal and mild-cognitive-impairment participants.

The original ADNI dataset had 2419 participants, comprising 895 cognitively normal (CN), 1113 mildly cognitively impaired (MCI), and 411 AD participants. A total of 2125 participants were excluded as they did not have CSF TGF-β isoforms measured. The baseline dataset comprised 294 participants with measurements of all three CSF TGF-β isoforms: 87 CN participants, 129 participants with MCI, and 78 participants with AD. Outcome-specific sample sizes differed because repeated-measure and complete covariate data were not available for every participant. The participant flow and analysis sets are presented in Figure 1.

The longitudinal models included 294 participants contributing 996 CSF Aβ observations, 231 participants contributing 649 observations for CSF total tau and CSF p-tau, 154 participants contributing 506 FDG-PET observations, 223 participants contributing 871 hippocampal-volume observations, 275 participants contributing 1350 ADAS-Cog 13 observations, and 275 participants contributing 1351 RAVLT immediate-recall observations. Conversion analyses used the follow-up dataset: 206 CN or MCI participants with 98 conversion events were included in both the logistic and time-to-event analyses.

### 3.2. Patient Demographics

The ADNI cohort comprised 87 CN, 129 MCI, and 78 AD participants (Table 1). Age and sex distribution were comparable across diagnostic groups. As expected, APOE ε4 carriers increased progressively from CN to AD. Baseline CSF Aβ42 levels were highest in CN and lowest in AD, while baseline CSF total tau and phosphorylated tau showed stepwise increases across the disease spectrum, consistent with established AD biomarker trajectories [22].

Individuals who demonstrated disease progression were labelled converters (*n* = 98, mean age ± SD was 75 ± 7 years, 33 females [34%]), while those who remained stable were labelled non-converters (*n* = 109, mean age ± SD was 75 ± 7 years, 49 females [45%]).

### 3.3. No Significant Differences in Baseline CSF TGF-β Isoforms Across CN, MCI, and AD Groups

All three TGF-β isoforms showed evidence of non-normal distributions (Shapiro–Wilk *p* < 0.001 for each isoform). Levene’s tests did not indicate heterogeneity of variance across diagnostic groups (TGF-β1 *p* = 0.941; TGF-β2 *p* = 0.622; TGF-β3 *p* = 0.452). Kruskal–Wallis tests showed no difference across CN, MCI, and AD groups for TGF-β1 (H = 0.071, *p* = 0.965), TGF-β2 (H = 2.594, *p* = 0.273), or TGF-β3 (H = 2.588, *p* = 0.274; Supplementary Figure S1).

Covariate-adjusted multiple linear regression analyses revealed no significant differences in CSF TGF-β isoform levels across diagnostic groups (CN, MCI, and AD). Specifically, diagnostic status was not associated with TGF-β1 (F [2254] = 0.775, *p* = 0.462; *q* = 0.667), TGF-β2 (F [2254] = 1.950, *p* = 0.145; *q* = 0.314), or TGF-β3 (F [2254] = 1.427, *p* = 0.242; *q* = 0.449) after adjustment for age, sex, APOE ε4 status, and baseline CSF Aβ.

### 3.4. Baseline CSF TGF-β1 Was Associated with CSF Tau Biomarkers

**Figure 2:**
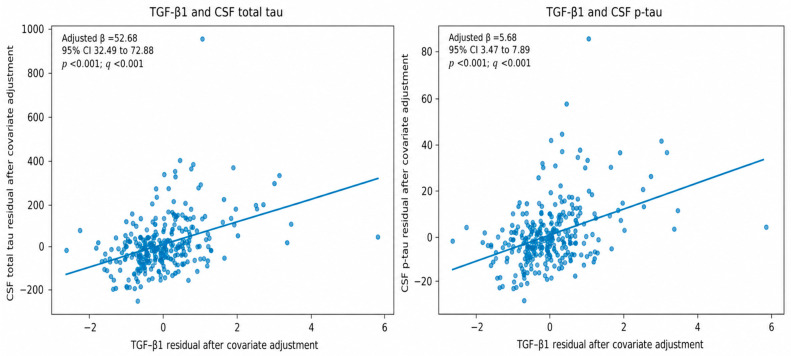
Covariate-adjusted associations of baseline CSF TGF-β1 with tau biomarkers. Partial-residual plots show the relationships of baseline TGF-β1 with total tau and p-tau after adjustment for age, sex, APOE ε4 carrier status, baseline diagnostic group, and Aβ42. Each point represents one participant. Higher TGF-β1 was associated with higher total tau (β = 52.68 pg/mL per 1 SD increase; 95% CI 32.49 to 72.88; *q* < 0.001) and higher p-tau (β = 5.68 pg/mL; 95% CI 3.47 to 7.89; *q* < 0.001).

In adjusted CSF biomarker models (Figure 2), higher baseline TGF-β1 was associated with higher total tau (β = 52.68 pg/mL per 1 SD; 95% CI 32.49 to 72.88; *p* < 0.001; *q* < 0.001) and higher p-tau (β = 5.68 pg/mL per 1 SD; 95% CI 3.47 to 7.89; *p* < 0.001; *q* < 0.001). Neither TGF-β2 nor TGF-β3 was associated with total tau or p-tau after FDR adjustment.

Higher TGF-β1 was associated with higher Aβ42 in the primary boundary-coded model (β = 60.72 pg/mL; 95% CI 13.22 to 108.23; *p* = 0.012; *q* = 0.037). However, this association was not retained when observations recorded at the Aβ42 assay ceiling were excluded (β = 36.14 pg/mL; 95% CI −1.68 to 73.96; *p* = 0.061; *q* = 0.183). In contrast, the TGF-β1 associations with total tau and p-tau remained statistically significant after excluding assay-limit values (total tau: β = 47.31, *q* < 0.001; p-tau: β = 5.18, *q* < 0.001). Thus, the robust baseline finding was the association of TGF-β1 with tau-related biomarkers rather than with Aβ42 (Supplementary Figure S2).

TGF-β2 and TGF-β3 were not associated with baseline Aβ42, total tau, or p-tau after correction for multiple testing. In addition, none of the three isoforms showed a significant association with baseline FDG-PET SUVR, hippocampal volume, RAVLT immediate recall, or ADAS-Cog 13 score after FDR correction.

### 3.5. Longitudinal CSF Biomarker Trajectories

Higher baseline TGF-β1 was nominally associated with a greater annual decline in Aβ42 (β = −8.21 pg/mL/year per 1-standard-deviation higher TGF-β1; 95% confidence interval [CI], −16.39 to −0.03; *p* = 0.049), but this association did not remain significant after FDR correction (q = 0.443). TGF-β2 (β = 3.03 pg/mL/year; 95% CI, −5.18 to 11.23; *p* = 0.470; *q* = 0.704) and TGF-β3 (β = −4.49 pg/mL/year; 95% CI, −12.70 to 3.71; *p* = 0.283; *q* = 0.704) were not associated with Aβ42 trajectory.

No significant association was observed for TGF-β1 (β = −2.32 pg/mL/year; 95% CI, −5.54 to 0.89; *p* = 0.157; *q* = 0.704), TGF-β2 (β = 0.15 pg/mL/year; 95% CI, −2.39 to 2.70; *p* = 0.907; *q* = 0.907), or TGF-β3 (β = −1.21 pg/mL/year; 95% CI, −4.24 to 1.82; *p* = 0.435; *q* = 0.704).

No significant association was observed for TGF-β1 (β = −0.17 pg/mL/year; 95% CI, −0.52 to 0.19; *p* = 0.355; *q* = 0.704), TGF-β2 (β = 0.06 pg/mL/year; 95% CI, −0.23 to 0.35; *p* = 0.700; *q* = 0.789), or TGF-β3 (β = −0.07 pg/mL/year; 95% CI, −0.41 to 0.28; *p* = 0.701; *q* = 0.789).

### 3.6. Longitudinal Neuroimaging and Cognitive Analyses

Baseline TGF-β1 was not associated with the longitudinal FDG-PET trajectory on mixed-effect analysis (time-by-TGF-β1 β = −0.005 SUVR units/year; 95% CI −0.015 to 0.004; *p* = 0.279; *q* = 0.425). TGF-β2 and TGF-β3 were not associated with FDG-PET slopes after FDR adjustment either.

**Figure 3:**
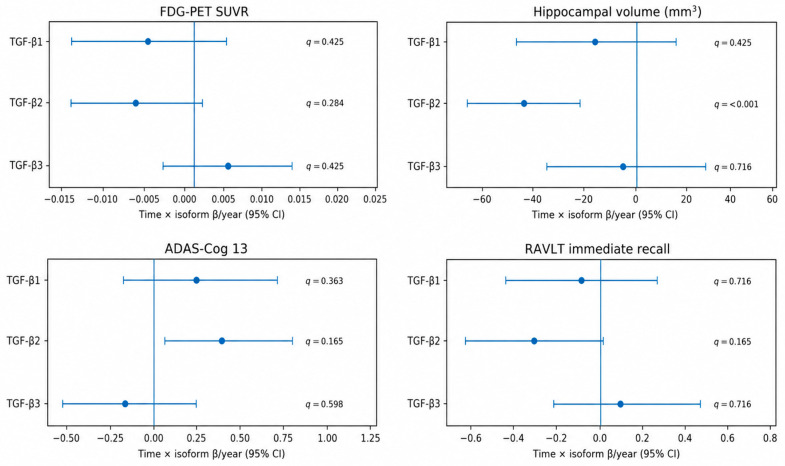
Associations of baseline CSF TGF-β isoforms with longitudinal imaging and cognitive trajectories. Forest plots display the time-by-isoform interaction from linear mixed-effect models, expressed as the additional annual change in each outcome per 1-standard-deviation higher baseline isoform concentration. Models included random intercepts and random slopes for time and adjusted for age, sex, APOE ε4 carrier status, baseline diagnosis, and Aβ42; hippocampal-volume models additionally adjusted for intracranial volume. The TGF-β2 association with hippocampal-volume decline remained significant after false-discovery-rate correction (β = −45.42 mm^3^/year; 95% CI −67.62 to −23.22; *q* = 0.001).

Higher baseline TGF-β2 was associated with a greater rate of hippocampal-volume loss (time-by-TGF-β2 β = −45.42 mm^3^/year; 95% CI −67.62 to −23.22; *p* < 0.001; *q* = <0.001) after adjustment for age, sex, APOE ε4 carrier status, baseline diagnostic group, Aβ42, and intracranial volume. No FDR-significant association with hippocampal slope was observed for TGF-β1 or TGF-β3. None of the isoforms was associated with longitudinal ADAS-Cog 13 or RAVLT immediate-recall change after FDR correction (Figure 3).

### 3.7. Clinical Conversion and Time-to-Event Analyses

In logistic-regression models, none of the isoforms showed an FDR-significant association with conversion status. The odds ratios per 1 SD higher isoform concentration were 1.40 for TGF-β1 (95% CI 0.98 to 2.01; *p* = 0.065; *q* = 0.097), 1.46 for TGF-β2 (95% CI 1.02 to 2.07; *p* = 0.038; *q* = 0.097), and 0.96 for TGF-β3 (95% CI 0.70 to 1.30; *p* = 0.784; *q* = 0.784).

**Figure 4:**
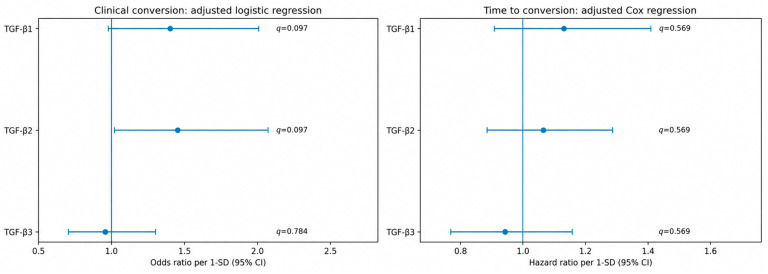
Baseline CSF TGF-β isoforms and clinical conversion. The left panel shows adjusted odds ratios for clinical conversion from logistic-regression models. The right panel shows adjusted hazard ratios for time to conversion from Cox proportional-hazard models stratified by baseline diagnostic group. Estimates are reported per 1-standard-deviation higher baseline TGF-β isoform concentration. No association remained significant after false-discovery-rate adjustment.

Time-to-event analyses likewise did not identify a significant association between any baseline isoform and conversion risk. The hazard ratios were 1.13 for TGF-β1 (95% CI 0.91 to 1.41; *p* = 0.272; *q* = 0.569), 1.07 for TGF-β2 (95% CI 0.88 to 1.28; *p* = 0.502; *q* = 0.569), and 0.94 for TGF-β3 (95% CI 0.77 to 1.16; *p* = 0.569; *q* = 0.569). Schoenfeld-residual diagnostics did not indicate a residual-time association for the isoform terms (Figure 4).

**Figure 5:**
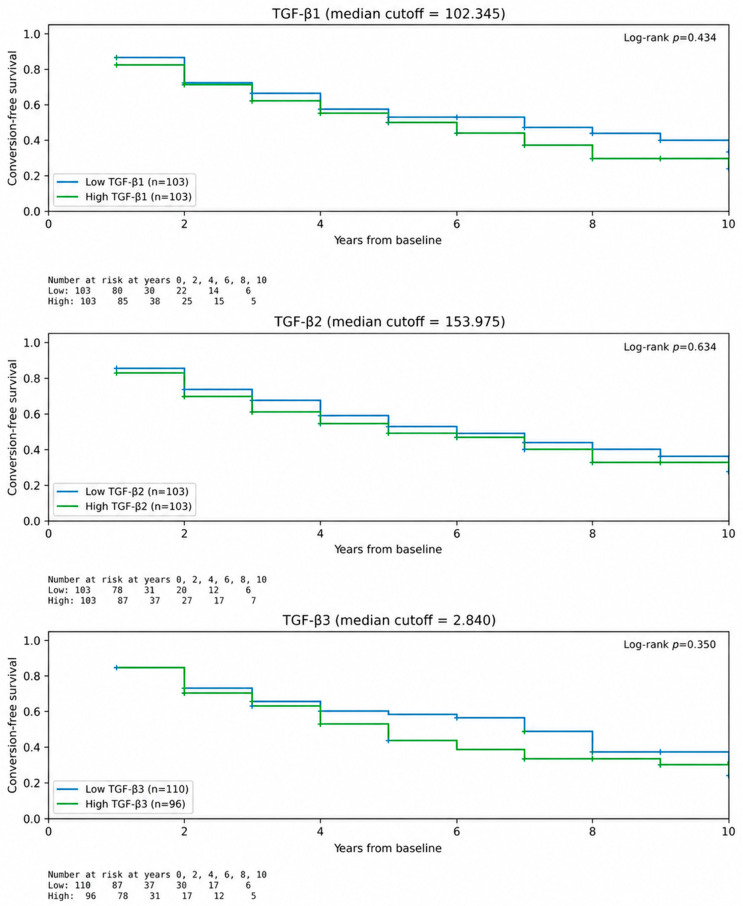
Kaplan–Meier estimates of conversion-free survival by baseline CSF TGF-β isoform level. Participants were classified as having baseline TGF-β1, TGF-β2, or TGF-β3 concentrations below or above the median. Blue curves represent the lower-concentration groups and green curves represent the higher-concentration groups. Censoring is indicated by plus symbols, with blue plus symbols denoting censored participants in the lower-concentration groups and green plus symbols denoting censored participants in the higher-concentration groups. Numbers at risk are displayed below each panel. Unadjusted log-rank tests showed no significant difference in conversion-free survival for TGF-β1 (*p* = 0.434), TGF-β2 (*p* = 0.634), or TGF-β3 (*p* = 0.350).

Kaplan–Meier analyses were concordant with the adjusted Cox models. Conversion-free survival did not differ between the lower and higher median-split groups for TGF-β1 (log-rank *p* = 0.434), TGF-β2 (*p* = 0.634), or TGF-β3 (*p* = 0.350; Figure 5).

## 4. Discussion

This study indicates that CSF TGF-β isoforms may be associated with distinct biological features of AD rather than functioning as interchangeable inflammatory markers. Higher baseline CSF TGF-β1 was robustly associated with higher baseline total tau and p-tau concentrations, whereas higher baseline TGF-β2 was associated with faster longitudinal hippocampal-volume loss. In contrast, none of the isoforms showed a robust relationship with baseline FDG-PET, cognitive performance, longitudinal FDG-PET decline, cognitive decline, clinical conversion, or time to conversion. These findings suggest that TGF-β signalling may be related to specific pathological and neurodegenerative processes without providing a reliable marker of clinical progression.

The strongest association was observed between TGF-β1 and tau-related CSF biomarkers. Higher baseline TGF-β1 was associated with both total tau and p-tau after adjustment for demographic factors, APOE ε4 status, baseline diagnosis, and CSF Aβ42. These associations remained significant after FDR correction and after exclusion of observations recorded at biomarker assay limits. Since total tau is generally interpreted as a marker of neuronal or axonal injury, whereas p-tau more closely reflects AD-related tau phosphorylation and aggregation, the concurrent association with both markers suggests that TGF-β1 may be linked to tau-associated neuronal stress or injury. However, the absence of significant longitudinal CSF biomarker associations argues against interpreting baseline CSF TGF-β isoforms as reliable indicators of future amyloid or tau biomarker dynamics.

Neuroinflammation is increasingly recognised as a central component of AD pathogenesis, involving interactions between microglia, astrocytes, endothelial cells, peripheral immune mediators, and adaptive immune responses. TGF-β1 is traditionally regarded as an anti-inflammatory cytokine because it can suppress excessive immune activation and support tissue homeostasis. Experimental studies have shown that exogenous TGF-β1 administration can reduce inflammatory signalling in models of amyloid-related injury, including lower microglial and T-cell-associated cytokine activity, reduced expression of TNF, IL-1β, inducible nitric oxide synthase, IL-17, and interferon γ, and preservation of neurotrophic or anti-inflammatory factors such as BDNF, IGF-1, GDNF, and IL-10. Experimental TGF-β1 supplementation has also been associated with reduced amyloid burden, reduced apoptosis, and improved memory performance in some AD-like models [6,15,16,18,23,24].

These experimental observations are not inconsistent with the present findings, but they suggest that the biological effects of TGF-β1 are likely context-dependent. Acute enhancement of TGF-β1 signalling may restrain damaging inflammatory responses, whereas chronically elevated endogenous CSF TGF-β1 may instead reflect persistent glial activation, neuronal injury, or an insufficient compensatory response to accumulating pathology. In this context, higher CSF TGF-β1 may not indicate successful neuroprotection. Rather, it may mark an ongoing state of immune and tissue-response signalling occurring alongside tau-related neuronal injury.

The relationship between TGF-β1 and tau biomarkers was more consistent than its relationship with amyloid. Although TGF-β1 showed a nominal association with higher CSF Aβ42 in the primary cross-sectional model, this association did not survive FDR correction and was not retained after exclusion of observations at the Aβ42 assay ceiling. It appears that TGF-β1 is associated more closely with tau-related pathology than with amyloid burden in this cohort. This pattern may be compatible with a model in which TGF-β1-related immune signalling becomes more prominent downstream of amyloid accumulation, at stages characterised by neuronal stress, synaptic dysfunction, and tau-associated injury.

TGF-β signalling is initiated when a ligand binds to type II TGF-β receptor dimers, followed by recruitment of type I receptors and formation of an active receptor complex. Canonical signalling involves phosphorylation of Smad2/3, formation of complexes with Smad4, and nuclear regulation of genes involved in cellular homeostasis and immune regulation. TGF-β receptors can also activate non-canonical pathways, including ERK, JNK, and PI3K signalling [11,19,25,26]. In chronic disease states, it has been proposed that the balance between these pathways may change, potentially reducing protective Smad-mediated responses while favouring signalling programs associated with inflammation, cellular stress, or cytotoxicity. Such mechanisms could theoretically contribute to impaired amyloid handling, altered tau phosphorylation, or maladaptive glial responses. However, the current analyses did not measure receptor abundance, Smad signalling, MAPK activity, or cell-specific TGF-β signalling. These mechanisms should therefore be viewed as biologically plausible hypotheses rather than direct explanations of the observed CSF associations.

The longitudinal association between higher baseline TGF-β2 and faster hippocampal-volume loss suggests a potentially different role for this isoform. TGF-β2 is highly expressed in the central nervous system and has been implicated in neuronal–glial communication, synaptic maintenance, cellular differentiation, and tissue homeostasis [27]. A relationship between baseline TGF-β2 and subsequent hippocampal atrophy may therefore indicate that altered TGF-β2 signalling accompanies increased vulnerability of medial temporal lobe structures. The hippocampus is especially susceptible to AD-related degeneration, and progressive hippocampal atrophy is strongly linked to memory impairment and disease advancement [28].

Nevertheless, this result should not be interpreted as evidence that TGF-β2 directly causes hippocampal degeneration. Elevated CSF TGF-β2 could represent an early response to structural injury, altered astrocyte-neuron signalling, or a broader neuroimmune state associated with hippocampal vulnerability. The lack of a significant cross-sectional relationship with baseline hippocampal volume may indicate that TGF-β2 is more closely related to dynamic structural change than to the degree of established atrophy at a single assessment. This interpretation requires replication in independent cohorts and would be strengthened by studies measuring longitudinal TGF-β2 changes alongside regional neuroimaging outcomes.

The absence of a robust association between TGF-β isoforms and FDG-PET decline is also informative. FDG-PET reflects a complex combination of synaptic activity, neuronal integrity, regional metabolic demand, and compensatory network function [29]. Baseline CSF TGF-β levels may not capture the mechanisms that determine longitudinal metabolic decline, even if they are related to inflammation or neurodegeneration in other ways.

Similarly, none of the TGF-β isoforms showed a robust association with longitudinal cognitive change after correction for multiple testing. Clinical performance is influenced by pathology, cognitive reserve, educational attainment, vascular disease, co-pathology, compensatory network mechanisms, and measurement variability [30,31]. It is therefore plausible that an inflammatory or injury-related CSF marker may relate to biological pathology without demonstrating a direct relationship with the subsequent rate of cognitive decline in a heterogeneous clinical cohort.

The conversion analyses provided no evidence that baseline CSF TGF-β isoforms predict transition to a more advanced clinical diagnosis. Neither logistic regression nor Cox proportional-hazard models supported a robust association between TGF-β1, TGF-β2, or TGF-β3 and clinical conversion. Kaplan–Meier analyses were consistent with these adjusted models. Clinical conversion is a threshold-based outcome influenced by factors beyond core AD pathology, including reserve, comorbidity, diagnostic uncertainty, and duration of observation [32]. Consequently, the absence of a conversion association does not negate the biological associations with tau biomarkers or hippocampal atrophy, but it does indicate that baseline CSF TGF-β isoforms should not be interpreted as prognostic clinical-conversion biomarkers in this cohort.

The receptor context of TGF-β signalling may also be relevant. TGFBR3, also known as betaglycan, regulates ligand availability and receptor signalling. Experimental evidence suggests that altered TGFBR3 expression may worsen AD-like pathology, including amyloid accumulation, synaptic loss, neuronal apoptosis, cognitive impairment, and pro-inflammatory microglial polarisation [33]. Similarly, suppression of the TGF-β1/CCN2/NF-κB pathway in APP/PS1 mice has been associated with reduced astrocyte-mediated inflammation, lower amyloid burden, and improved cognitive performance [34]. Together, these observations support the possibility that the functional consequence of TGF-β signalling depends not only on ligand concentration, but also on receptor availability, cellular source, disease stage, and downstream pathway activation. Elevated CSF TGF-β1 may therefore reflect altered pathway engagement rather than effective anti-inflammatory signalling. However, this interpretation remains speculative, because receptor and downstream signalling measures were not available in the present dataset.

The study has several strengths. All three TGF-β isoforms were examined as prespecified candidate biomarkers in parallel rather than selecting one isoform on the basis of nominal statistical findings. The analyses used a fixed covariate strategy and avoided principal component analysis or backward-selection procedures for biomarker prioritisation. Repeated outcomes were analysed using mixed-effect models, and the findings are reported with effect estimates, confidence intervals, sample sizes, and FDR-adjusted *q* values. These features improve transparency and reduce the risk of selective interpretation.

Several limitations should be considered. The study was observational and therefore cannot establish causal direction. TGF-β isoforms were measured only at baseline, making it impossible to determine whether within-person changes in TGF-β signalling track changes in tau, amyloid, hippocampal atrophy, cognition, or clinical status. CSF concentrations may not fully represent region-specific or cell-specific signalling activity in the brain. Potential influences of systemic inflammation, medication exposure, vascular disease, peripheral TGF-β sources, and additional inflammatory mediators were not comprehensively measured. In addition, some longitudinal analyses had smaller samples because repeated data were unavailable for all participants. Finally, the ADNI cohort is highly selected and the findings require replication in independent and more diverse cohorts using harmonised assays and repeated CSF sampling.

In conclusion, the findings support an isoform-specific interpretation of CSF TGF-β signalling in AD. Higher baseline TGF-β1 was associated with higher total tau and p-tau, while higher baseline TGF-β2 was associated with faster hippocampal-volume loss. These findings may reflect distinct relationships between TGF-β signalling, tau-related neuronal injury, and structural neurodegeneration. However, baseline TGF-β isoforms were not robustly associated with longitudinal FDG-PET decline, cognitive decline, clinical conversion, or time to conversion. CSF TGF-β isoforms should therefore be interpreted as potential biological correlates of disease-related pathology rather than established prognostic markers of clinical progression.

## Figures and Tables

**Table 1:** Demographic, genetic, biomarker, neuroimaging, and cognitive characteristics of the ADNI population.

	Cognitively Normal	Mild Cognitive Impairment	Alzheimer’s Disease	*p*
*n*	87	129	78	
Age, years (mean ± SD)	75.7 ± 5.45	74.3 ± 7.72	74.9 ± 8.19	0.382
Sex (female, %)	39 (45%)	48 (37%)	35 (45%)	0.419 *
APOEε4 carriers (*n*, %)	22 (25%)	69 (53%)	55 (71%)	2.5 × 10^−8^ *
CSF Aβ_42_ (pg/mL, mean ± SD)	1186 ± 426	868 ± 436	642 ± 327	3.1 × 10^−15^
CSF total tau (pg/mL, mean ± SD)	234 ± 85.3	310 ± 142	357 ± 136	5.7 × 10^−9^
CSF phosphorylated tau (pg/mL, mean ± SD)	21.5 ± 8.7	30.2 ± 15.2	36.3 ± 16.2	2.6 × 10^−10^
FDG-PET SUVR	1.28 ± 0.14	1.16 ± 0.14	1.06 ± 0.11	<1 × 10^−15^
Hippocampal volume (mm^3^)	7253 ± 768	6261 ± 1142	5830 ± 1101	<1 × 10^−15^
RAVLT	42.7 ± 8.9	30.1 ± 8.8	23.1 ± 7.6	<1 × 10^−15^
ADAS-Cog 13	9.9 ± 4.3	18.7 ± 6.2	28.8 ± 7.9	<1 × 10^−15^

One-way ANOVA was used for most of the measures, except for * chi-squared test used for computing *p* values for sex and APOEε4 carriers.

## Data Availability

Data used in preparation of this article were obtained from the ADNI database (http://adni.loni.usc.edu/, accessed on 20 April 2026). As such, ADNI investigators contributed to study design and implementation and provided data, but did not participate in analysis or writing of this report. For a complete listing of ADNI investigators, see https://adni.loni.usc.edu/wp-content/uploads/how_to_apply/ADNI_Acknowledgement_List.pdf (accessed on 20 April 2026). ADNI data are disseminated by the Laboratory of Neuroimaging at the University of Southern California. All ADNI data are available for public access at adni.loni.usc.edu contingent on adherence to the ADNI Data Use Agreement.

## Supplementary Materials

The following supporting information can be downloaded at https://www.mdpi.com/article/10.3390/neurolint18070138/s1. Figure S1: Baseline CSF TGF-β isoform concentrations by diagnostic group. Box plots and individual data points show baseline CSF TGF-β1, TGF-β2, and TGF-β3 concentrations in cognitively normal, mild cognitive impairment, and Alzheimer’s disease groups. No diagnostic-group differences were identified by Kruskal-Wallis testing: TGF-β1, *H* = 0.071, *p* = 0.965; TGF-β2, H = 2.594, *p* = 0.273; TGF-β3, H=2.588, *p* = 0.274. Figure S2: Adjusted associations of baseline CSF TGF-β isoforms with established Alzheimer’s disease CSF biomarkers. Forest plots show adjusted regression coefficients and 95% confidence intervals per 1-standard-deviation higher baseline isoform concentration. Aβ42 models were adjusted for age, sex, APOE ε4 carrier status, and baseline diagnosis. Total tau and p-tau models additionally adjusted for baseline Aβ42. *q* values were calculated using the Benjamini-Hochberg false-discovery-rate procedure across the nine isoform-biomarker tests. TGF-β1 was robustly associated with total tau and p-tau.
