# Supplementary material for: Cerebrospinal Fluid Transforming Growth Factor β Isoforms and Disease Progression in Alzheimer’s Disease: Longitudinal Evidence from the ADNI Cohort
Source: Neurol Int. 2026 Jul 20;18(7):138. doi: 10.3390/neurolint18070138 (PMC13415252; doi:10.3390/neurolint18070138)
Supplement: Supplementary file 1 [file neurolint-18-00138-s001.zip › neurolint-4333889-supplementary.pdf]

Supplementary Materials and Methods

ADNI Study

The collection of CSF measurements, neuroimaging, and psychological assessments were previously collected by ADNI researchers and were not the authors' work. The methodology outline is a repeat of ADNI's own procedures. The present study represents a secondary analysis of this publicly available dataset (further information available at adni.loni.usc.edu).

The data used for the preparation of this article were downloaded in September 2024 from the ADNI database (http://adni.loni.usc.edu/) – ADNI 1 and ADNI GO. ADNI-2 and 3 were excluded as CSF measures for proteins of interest were not available. The ADNI study has been previously described in detail[1]. Written informed consent was obtained from the patients/participants for the publication of any identifiable data included in this article. Participants were genotyped for apolipoprotein E (APOE) ε4. The Alzheimer's Disease Neuroimaging Initiative (ADNI) utilizes clinical and neuropsychological assessments, imaging and CSF biomarkers over time to track progression of the disease. The use of human data in ADNI database was approved by the institutional review board in participating centres and written informed consent was obtained. All studies in human participants were reviewed and approved based on ADNI protocols, all procedures involving human participants were approved by the institutional and/or national research committee, or by the 1964 Helsinki declaration and its amendments, or comparable ethical standards.

CSF measurements for TGF-β1, TGF-β2, and TGF-β3

CSF analysis was performed by two skilled research scientists experienced in multiplex assays blinded to diagnosis and other subject-level information. All samples were run in duplicate with six CSF standards on each plate, and CSF inflammatory protein levels were normalized across plates using the six CSF standard values. ADNI CSF samples were first randomized across twelve 96-well plates, and each batch was analysed for levels of proteins during the same two-day block to avoid freeze-thawing. All samples were run in duplicate with six CSF standards on each plate. CSF inflammatory protein levels were normalized across plates using the six CSF standard values, and intermediate precision for each analyte was then calculated using inter-plate coefficient of variation: 8.62% for TGF-β1, 7.62% for TGF-β2, and 7.70% for TGF-β3.

CSF measurements for Aβ, total tau, and p-tau

CSF samples were obtained in the morning following overnight fasting at the baseline visit. The time from collection to freezing was ~1 h, with processing, aliquoting and storage at −80°C as per ADNI Biomarker Core Laboratory Standard Operating Procedures. CSF Aβ1−42, total tau and p-tau were measured using the Luminex platform as described previously[1].

Inclusion/Exclusion Criteria

Those enrolled in the ADNI-1 cohort were aged 55–90 years, had a study partner capable of independently providing a rating of the recruited participant's functioning, and spoke fluent English or Spanish. For this sample, participants needed to have a Hachinski Ischaemic score ≤4, geriatric depression scale <6, with visual and auditory acuity sufficient for neuropsychological testing, sixth grades education or work history and not in any other trials or studies. Other exclusion criteria included subjects on specific psychoactive drugs e.g., narcotic analgesics, neuroleptics, anticholinergic agents and antiparkinsonian drugs, all investigational drugs, benzodiazepines, and frequently central nervous system active antihypertensive agents, antidepressants, within 4 weeks before screening. Patients with any other significant neurological illness apart from AD, a history of brain lesions or traumatic brain injury, were excluded as well.

Cognitively normal (CN) participants must show no significant impairment in any of the cognitive domains assessed, impaired activities of daily living, a mini-mental state examination (MMSE) score between 24 and 30, a CDR of 0, be not depressed, and not have mild cognitive impairment (MCI) or dementia. CN subjects were age range-matched to both MCI and AD subjects. MCI patients need to have an MMSE score of 24–30, report a memory complaint, show objective memory loss (education adjusted scores on Wechsler Memory Scale Logical Memory II) below the cut-off, a CDR of 0.5 (however, no observed impairment in other cognitive domains and preserved activities of daily living), and no dementia. The AD cases in this study had MMSE scores ranging from 20 to 26, CDR of 0.5 or 1.0 and fulfilled National Institute of Neurological and Communicative Disorders and Stroke-Alzheimer's Disease and Related Disorders Association criteria for probable AD (NINDS–ADRDA).

Tracking disease progression

Tracking disease progression is a primary outcome measure of the ADNI protocol. Site physicians review data from each participant and complete diagnostic summary. If a clinician incurs a change in diagnosis (e.g., from CN to MCI or from MCI to AD), the neuropsychological assessments (e.g., CDR-SB) for that visit are reviewed by the onsite clinical monitor. If the site's primary investigator attempts to raise the clinical monitor, the clinical monitor should have the power to challenge them and direct the diagnosis to be reversed if reported incorrectly. The ADNI clinical co-investigator then evaluates the data and instructs the clinical monitor to clarify any discrepancies in scoring. ADNI conversion committee is then tasked with reviewing all the patient reports when this review is finalized and as per the NINDS-ARCDA (general procedures manual explain in http://adni.loni.usc.edu/), a consensus is achieved on the conversion status of participant. Though ultimately a neuropathological diagnosis remains essential to diagnosis of AD, high sensitivity and specificity has been demonstrated for the application of neuroimaging and neuropsychological assessments that in many cases are sufficient to determine disease progression towards AD[2-4].

Structural MRI Volumes

Subjects were scanned at 1.5T with a 3D sagittal volumetric magnetization prepared rapid gradient echo (MP-RAGE) sequence[5]. Acquisition parameters were repetition time, 9 ms; echo time, 4 ms; flip angle 8° along with a 256 × 256 × 170 acquisition

matrix in the x-, y- and z-dimensions yielding a nominal voxel size of 0.94 × 0.94 × 1.2 mm^3. MRI was performed at baseline, 6 months, 1 year, and yearly for 6 years thereafter. Hippocampal volumes were derived using FreeSurfer (version 4.1.0) and as previously described[6]. MRI volumetric images were motion corrected, non-brain tissue was removed using hybrid watershed or surface deformation, the images were Talairach transformed automatically, and subsequently subcortical white and deep grey matter structures were segmented. Subsequently, the intensity normalization, tessellation of grey and white matter boundary and automated topology correction. The signal intensities of the hippocampus and amygdala are similar, but their spatial location is consistent with each other in that the amygdala is always anterior to and superior to the hippocampus. The second part involved the use of (and modifications to) the Markov random field model, which ensured that the segmentations generated were anatomically plausible and that it was spatially non-stationary. This is done by independently modelling the probabilities of the hippocampus above and below the amygdala leading to an accurate capture of the hippocampus. Hippocampal volume was computed as the product of the number of hippocampal voxels and the voxel volume.

[^{18}F] Fluorodeoxyglucose ([^{18}F]FDG-PET)

[18F]FDG-PET was performed on multiple scanners with variable resolution at 6 months, and at 1, 1.5 and 2 years[7]. Scans were obtained in 6 × 5-min frames, starting 30 min after the intravenous administration of 5 mCi of 18F-FDG. (FDG-PET) images were pre-processed following standard ADNI procedures with frames co-registered, averaged and reoriented along the anterior-posterior commissure line and resliced to 1.5 mm isotropic voxel space. All PET images were spatially normalized to Montreal Neurological Institute (MNI) space, and the mean hippocampal FDG uptake (normalized to pons uptake) was determined.

Neuropsychological Assessments

Rey Auditory Verbal Learning Test (RAVLT) and the Alzheimer's Disease Assessment Scale-Cognitive Subscale 13-item version (ADAS-Cog 13) served as sensitive markers of progression. RAVLT measures episodic verbal memory by testing an individual's ability to learn 15 words across five trials. The test consisted of a short delay recall trial followed by a distracter list and then a 30 min long delay recall trial followed by a yes/no recognition trial (http://www.adni-info.org). The ADAS-Cog 13 is a 13-item derivative of the ADAS-Cog commonly used for the assessment of learning, memory, language production and comprehension, constructional and ideational praxis, orientation and contains number cancellation as well as delayed free recall tasks. The word recall test was split first, and the word recognition task at the end with other cognitive tasks in between. Because of the two-word memory tasks, the two groups were separated to minimize the risk of mixing words from the two tasks. The use of one or more objective tests was followed by subjective clinical evaluation of the laterality of language and short-term memory or the capacity of the participant in recalling the test instructions (more details available on adni.loni.usc.edu/wp-content/uploads/2010/09/ADNI_GeneralProceduresManual.pdf).

References

1 Weiner, M. W. *et al.* The Alzheimer's Disease Neuroimaging Initiative: a review of papers published since its inception. *Alzheimers Dement* **8**, S1-68, doi:10.1016/j.jalz.2011.09.172 (2012).

2 Petersen, R. C. *et al.* Alzheimer's Disease Neuroimaging Initiative (ADNI): clinical characterization. *Neurology* **74**, 201-209, doi:10.1212/WNL.0b013e3181cb3e25 (2010).

3 Davatzikos, C., Genc, A., Xu, D. & Resnick, S. M. Voxel-based morphometry using the RAVENS maps: methods and validation using simulated longitudinal atrophy. *Neuroimage* **14**, 1361-1369, doi:10.1006/nimg.2001.0937 (2001).

4 Fan, Y., Batmanghelich, N., Clark, C. M., Davatzikos, C. & Alzheimer's Disease Neuroimaging, I. Spatial patterns of brain atrophy in MCI patients, identified via high-dimensional pattern classification, predict subsequent cognitive decline. *Neuroimage* **39**, 1731-1743, doi:10.1016/j.neuroimage.2007.10.031 (2008).

5 Jack, C. R., Jr. *et al.* Update on the magnetic resonance imaging core of the Alzheimer's disease neuroimaging initiative. *Alzheimers Dement* **6**, 212-220, doi:10.1016/j.jalz.2010.03.004 (2010).

6 Fischl, B. *et al.* Whole brain segmentation: automated labeling of neuroanatomical structures in the human brain. *Neuron* **33**, 341-355, doi:10.1016/s0896-6273(02)00569-x (2002).

7 Jagust, W. J. *et al.* The Alzheimer's Disease Neuroimaging Initiative positron emission tomography core. *Alzheimers Dement* **6**, 221-229, doi:10.1016/j.jalz.2010.03.003 (2010).

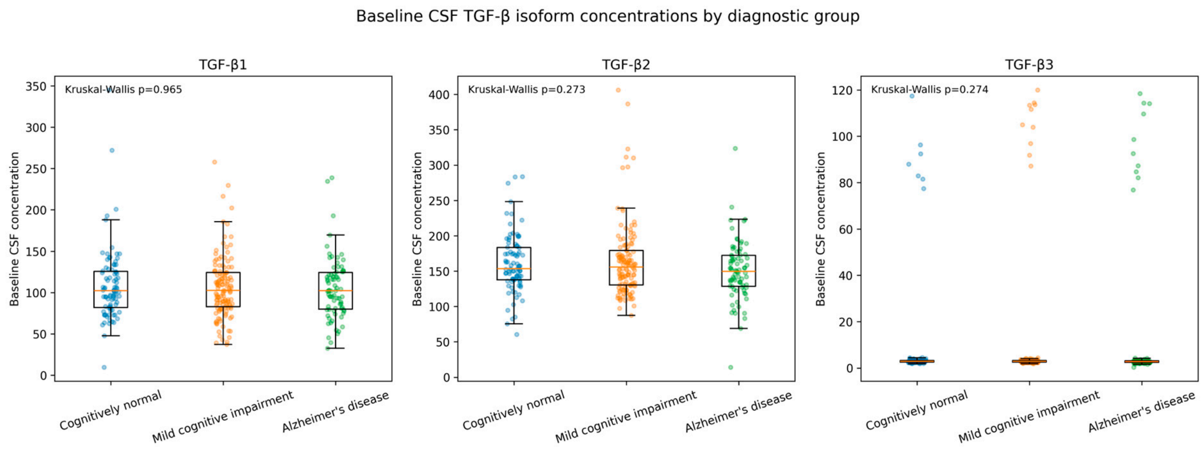

Supplementary Figure S1. Baseline CSF TGF-β isoform concentrations by diagnostic group. Box plots and individual data points show baseline CSF TGF-β1, TGF-β2, and TGF-β3 concentrations in cognitively normal, mild cognitive impairment, and Alzheimer's disease groups. No diagnostic-group differences were identified by Kruskal-Wallis testing: TGF-β1, H=0.071, p=0.965; TGF-β2, H=2.594, p=0.273; TGF-β3, H=2.588, p=0.274.

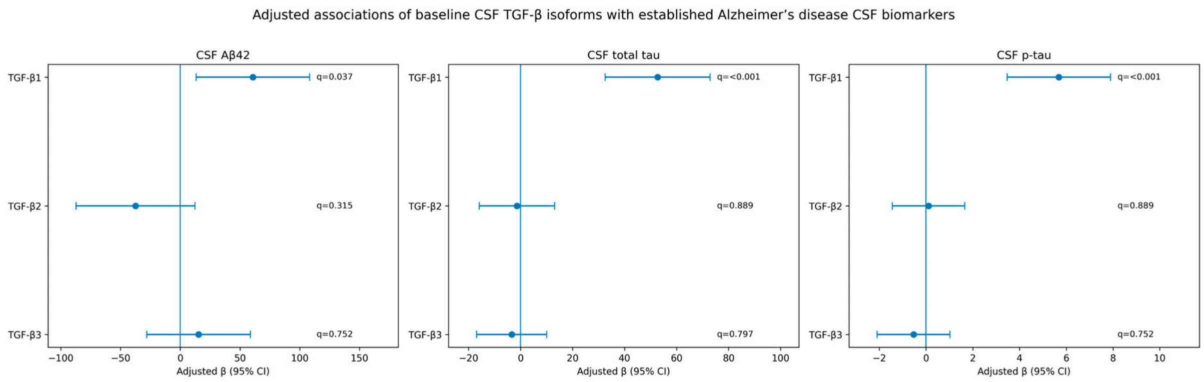

Supplementary Figure S2. Adjusted associations of baseline CSF TGF-β isoforms with established Alzheimer's disease CSF biomarkers. Forest plots show adjusted regression coefficients and 95% confidence intervals per 1-standard-deviation higher baseline isoform concentration. Aβ42 models were adjusted for age, sex, APOE ε4 carrier status, and baseline diagnosis. Total tau and p-tau models additionally adjusted for baseline Aβ42. q values were calculated using the Benjamini-Hochberg false-discovery-rate procedure across the nine isoform-biomarker tests. TGF-β1 was robustly associated with total tau and p-tau.
